# Draft Genome Sequence of Ammonia-Producing *Acinetobacter* sp. Strain MCC2139 from Dairy Effluent

**DOI:** 10.1128/genomeA.00410-13

**Published:** 2013-06-27

**Authors:** Debasmita Chatterjee, Ashoke Ranjan Thakur, Shaon RayChaudhuri

**Affiliations:** West Bengal University of Technology, Salt Lake, Kolkata, West Bengal, Indiaa; Techno India University, Salt Lake, Kolkata, West Bengal, Indiab

## Abstract

We report the draft genome sequence of an ammonia-producing, esculin-hydrolyzing, catalase-positive, gram-negative bacterium, *Acinetobacter* sp. strain MCC2139. This bacterium, isolated from dairy sludge and with optimum growth at 37°C, has a genome size of 2,967,280 bp with a G+C content of 42.3%.

## GENOME ANNOUNCEMENT

Ammonia is a fundamental chemical compound for the production of nitrogen-based fertilizers. The highly energy-intensive Haber-Bosch process is conventionally used for producing ammonia. Globally, 109 million tons of ammonia is produced annually, releasing 208 million tons of CO_2_. It accounts for 1.2% of the global primary energy demand (1). Approximately 75% of the ammonia produced is used as fertilizer, while the rest is used as raw material in the manufacture of polymeric resins, explosives, nitric acid, and other products.

Sludge from a dairy effluent treatment plant was used for the isolation of a bacterial strain, *Acinetobacter* sp. MCC2139, with potential for treating dairy effluent for the production of ammonia. The strain produced ammonia from whey water under unamended ambient conditions. It also removed 53.5% carbohydrate, 77.81% protein, 23.23% chloride, 44.44% calcium carbonate, 40.14% nitrate, 11.25% nitrite, and 17.69% phosphate from dairy effluent within 24 h with aeration (bubbling) under ambient conditions. The strain is available at the Microbial Culture Collection at the National Centre for Cell Sciences, Pune, India, with the accession number MCC2139.

The genome sequencing was carried out on an Ion Torrent PGM instrument using a 316 chip. The total assembled reads (using MIRA Assembler version 3.4.0) were 1,435,352 bp, with 69.01× coverage. There were 141 contigs. A total of 29.67 Mb of data was sequenced, with the largest contig size of 163,183 bp. All contigs generated were submitted to the RAST (Rapid Annotation using Subsystem Technology) (2) annotation server and the corresponding information on coding sequences (CDS) was generated.

The important genes encoded by the different contigs are as follows: genes for tRNA pseudouridine synthase A; transcriptional regulator (AraC family); phosphoserine phosphatize; 23S rRNA; Na(+) H(+) antiporter subunits; type IV fimbrial assembly (AOTJ01000001); copper chaperone as well as lead-, cadmium-, zinc- and mercury-transporting ATPase; Cu(I)-responsive transcriptional regulator (AOTJ0100009); ferric iron ATP-binding cassette (ABC) transporter; aromatic amino acid transport protein AroP(AOTJ0100008); cobalt-zinc-cadmium resistance protein CzcD (AOTJ0100076); chromosome (plasmid) partitioning proteins ParB and ParA (AOTJ0100068); chromate transport protein ChrA/resistant protein ChrB (AOTJ0100061); phage-related protein (AOTJ0100053); superoxide dismutase [Fe] (AOTJ0100056); rod-shape-determining protein MreB/MreC (AOTJ0100057); ABC1 family protein (AOTJ0100048); exonuclease SbcC/SbcD (AOTJ0100049); transcriptional regulator (LuxR family); Kup system potassium uptake protein (AOTJ0100047); Fe-S oxidoreductase (AOTJ0100045); inorganic pyrophosphatase; phosphate transport system regulatory protein PhoU; ADP-ribose pyrophosphatase; ammonium transporter; and nitrogen regulatory protein P–II (AOTJ0100042).

### Nucleotide sequence accession numbers.

This whole-genome shotgun project has been deposited at DDBJ/EMBL/GenBank under the accession number AOTJ00000000. The version described in this paper is the first version, AOTJ01000000.
